# The Lightfield Microscope Eyepiece

**DOI:** 10.3390/s21196619

**Published:** 2021-10-05

**Authors:** Nicolò Incardona, Ángel Tolosa, Gabriele Scrofani, Manuel Martinez-Corral, Genaro Saavedra

**Affiliations:** 1 3D Imaging and Display Laboratory, Department of Optics, Universitat de València, 46100 Burjassot, Spain; angel.tolosa@uv.es (Á.T.); gabriele.scrofani@uv.es (G.S.); manuel.martinez@uv.es (M.M.-C.); genaro.saavedra@uv.es (G.S.); 2 Doitplenoptic S.L., 46980 Paterna, Spain

**Keywords:** lightfield microscopy, Fourier integral microscope, FiMic, lightfield eyepiece, plenoptic eyepiece, 3D microscopy

## Abstract

Lightfield microscopy has raised growing interest in the last few years. Its ability to get three-dimensional information about the sample in a single shot makes it suitable for many applications in which time resolution is fundamental. In this paper we present a novel device, which is capable of converting any conventional microscope into a lightfield microscope. Based on the Fourier integral microscope concept, we designed the lightfield microscope eyepiece. This is coupled to the eyepiece port, to let the user exploit all the host microscope’s components (objective turret, illumination systems, translation stage, etc.) and get a 3D reconstruction of the sample. After the optical design, a proof-of-concept device was built with off-the-shelf optomechanical components. Here, its optical performances are demonstrated, which show good matching with the theoretical ones. Then, the pictures of different samples taken with the lightfield eyepiece are shown, along with the corresponding reconstructions. We demonstrated the functioning of the lightfield eyepiece and lay the foundation for the development of a commercial device that works with any microscope.

## 1. Introduction

Widefield microscopy is unsuitable for applications in which three-dimensional information of the sample is required, due to the small depth of field and the lack of optical sectioning. For this reason, many microscopy techniques have been developed to obtain 3D information. Many of them rely on capturing multiple images [1,2,3,4,5,6,7], which makes them inappropriate for moving samples and dynamic processes. Lightfield microscopy is an emerging technique that solves this problem thanks to its capacity to capture three-dimensional information in a single shot. This allows fast volume acquisition (essentially limited by the camera frame rate), enabling applications in which high time resolution is essential [8,9,10].

The lightfield technique proceeded from Integral Photography [11], and was firstly applied to microscopy at Stanford [12]. This first approach consisted in placing a microlens array (MLA) at the image plane of the microscope and a sensor at the back focal plane of the MLA. In this way, the pixels behind each microlens register angular information of one point of the sample, which is used to reconstruct 3D information. Recently, a new approach was developed, in which the MLA is placed at the Fourier plane of the microscope objective [13]. This new setup is called Fourier integral microscope (FiMic), or Fourier lightfield microscope (FLFM) in later developments [14]. As demonstrated in [13], this system provides better lateral resolution and depth of field than the first one. In particular, the lateral resolution is improved by a factor of $\sqrt{2}$ in the raw microimages, without need for digital image processing. Moreover, in the reconstruction, the lateral resolution is constant along the entire depth of field, and there is no artifact near the native image plane. Therefore, FiMic is establishing itself as the standard setup for lightfield microscopy [15,16,17,18].

**Figure 1:**
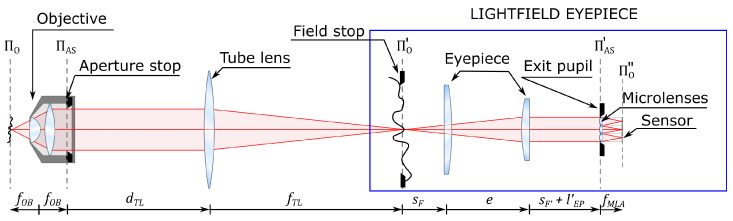
Optical scheme of the Fourier integral microscope with the lightfield microscope eyepiece highlighted in blue. Π_0_ is the object plane, $f_{\mathrm{OB}}$ is the focal length of the microscope objective, Π_AS_ is the plane of the aperture stop of the microscope objective. $d_{\mathrm{TL}}$ is the distance from the aperture stop plane to the tube lens, $f_{\mathrm{TL}}$ is the focal length of the tube lens. Π’_0_ is the conjugate of Π_0_, namely the intermediate image plane. $s_{F}$ is the distance from the front focal plane of the eypiece to the first lens of the eyepiece, *e* is the distance between the lenses of the eyepiece, $s_{F^{\prime }}$ is the distance from the second lens of the eyepiece to the back focal plane of the eyepiece. Π’_AS_ is the conjugate of Π_AS_, namely the exit pupil plane, ${l^{\prime }}_{\mathrm{EP}}$ is the distance from the back focal plane of the eyepiece to the exit pupil plane, $f_{\mathrm{MLA}}$ is the focal length of the microlenses. Π”_0_ is the conjugate of Π’_0_, namely the image plane.

The optical scheme of FiMic is presented in Figure 1. As it is not easy to insert a MLA at the aperture stop of the microscope objective, a relay is used to conjugate the aperture stop plane with the MLA plane. This geometry allows the insertion of a field stop to avoid image overlapping at the sensor. Looking at the optical scheme, we can see that the first three components (objective, tube lens, and eyepiece) form the setup of a conventional microscope. From this, we can deduce that the system composed of the eyepiece, the MLA, and the sensor (highlighted in blue) can be treated as a lightfield microscope eyepiece. By replacing the conventional eyepiece with the lightfield eyepiece, essentially every conventional microscope can be converted into a lightfield microscope. In this way, there is no need to construct the entire system on the optical table, and one can exploit all the components of the native microscope, such as the illumination system, translation stage, filter wheel, etc.

Here we demonstrate the design, construction, and functioning of this novel device. Then, we demonstrate that the experimental parameters agree with the theoretical ones and show some experimental images with different kinds of samples. For the experimental images, the device was attached to different microscopes, thus demonstrating its universality.

## 2. Materials and Methods

### 2.1. Parametrization

For the construction of the proof-of-concept device, we used a Nikon TE2000-U as a host microscope. We designed the device to adapt it to two routine objectives in terms of magnification and numerical aperture: a 20x/0.50 (CFI Plan Fluor 20X from Nikon) and a 40x/0.75 objective (UPlanFl N 40X from Olympus). It is important to underline that the device works exclusively with infinity-corrected objectives.

First of all, we had to choose the geometrical parameters of the components of the lightfield eyepiece, namely the focal length of the eyepiece lens (*f_EP_*), the pitch (*p_MLA_*), and focal length (*f_MLA_*) of the microlens array and the pixel size of the sensor (δ). We decided to work with a hexagonal-shaped MLA, with the central microlens aligned with the optical axis of the microscope. For this reason, we must fit at least 3 microlenses into the diameter of the exit pupil. This condition sets the first requirement:

(1) $$N=\frac{\Phi_{\mathrm{AS}}}{\frac{f_{\mathrm{TL}}}{f_{\mathrm{EP}}}p_{\mathrm{MLA}}}\geq 3,$$

where *N* is the number of microlenses that fit into the diameter of the exit pupil, Φ*_AS_* is the aperture stop diameter of the microscope objective and *f_TL_* is the focal length of the tube lens. These two parameters are set by the host microscope and the objective (Φ_*AS*_ = 6.75 mm for the 40× objective and *f_TL_* = 200 mm for Nikon microscopes). Moreover, we set a minimum pitch of 1 mm, because smaller microlenses would reduce too much the field of view ($FOV=\frac{p_{\mathrm{MLA}}}{M_{\mathrm{TOT}}}$, where *M_TOT_* is the total magnification of the entire FiMic system). If we solve Equation (1), putting a safety margin of 10% for *N*, we obtain *f_EP_* > 97.8 mm. So, we fixed *f_EP_* = 100 mm as the focal length of the eyepiece lens. With all these parameters, we obtain *N* = 3.375 for the 40x objective. As only the microlenses that fit entirely into the exit pupil can be considered useful, we have 3 useful microlenses in the diameter. In the 20x objective, Φ_*AS*_ = 10 mm, so we obtain *N* = 5.

We chose a fused silica MLA with $f_{\mathrm{MLA}}=7.92$ mm. This is a key parameter, as the total magnification of the system depends on it:

(2) $$M_{\mathrm{TOT}}=M_{\mathrm{OB}}\frac{f_{\mathrm{MLA}}}{f_{\mathrm{EP}}}.$$

Note that, when calculating the total magnification of the system with the 40x objective, the effective magnification of the considered objective is *M*_*OB,eff*_ = 44.4. In fact, the reference focal length of the tube lens for Olympus is 180 mm, while for Nikon it is 200 mm.

To avoid overlapping between microimages, we must match the f-number of the eyepiece lens with that of the microlenses. Thus, we add a field stop whose diameter, Φ*_FS_*, is such that

(3) $$\frac{\Phi_{\mathrm{FS}}}{f_{\mathrm{EP}}}<\frac{p_{\mathrm{MLA}}}{f_{\mathrm{MLA}}},$$

so we fixed $\Phi_{\mathrm{FS}}=12.5$ mm. As we chose a field stop diameter slightly lower than the maximum diameter permitted to avoid overlapping, the effective field of view is

(4) $$FOV=\frac{\Phi_{\mathrm{FS}}}{M_{\mathrm{OB},\mathrm{eff}}}.$$

We have *FOV* = 625 µm with the 20x objective and *FOV* = 281 µm with the 40x objective.

Finally, we chose the pixel size of the sensor. For this purpose, we matched the resolution limits given by diffraction and by the Nyquist theorem [13]:

(5) $$\frac{\lambda N}{2NA_{\mathrm{OB}}}=\frac{2\delta }{M_{\mathrm{TOT}}}.$$

Note that, in Equation (5), we consider that the numerical aperture of the illumination system is the same as the numerical aperture of the microscope objective. If we substitute into this equation the data of either objective (the result actually depends exclusively on the parameters of the MLA) and we consider a wavelength λ=600 nm, we obtain δ = 2.37 µm. So, we picked a CMOS sensor with 2.20 µm pixel size, which is very close to the required value. As we chose a pixel size lower than that calculated in Equation 5, the resolution is limited by diffraction. We have ρ = 3 µm for the 20x objective and ρ = 1.35 µm for the 40x objective, where ρ is the resolution limit.

### 2.2. Optical Design

For the eyepiece, we decided to use the Ramsden design. The decision to use a combination of lenses instead of a single lens is due to two reasons. The first one is to reduce aberrations, in fact, the Ramsden eyepiece reduces chromatic aberrations, spherical aberrations, distortion, astigmatism, and coma [19]. The second reason is that this combination reduces the distance between the focal planes of the eyepiece, thus decreasing the total physical length of the device. This is a critical parameter because an excessive length of the device would produce severe tilt when inserted in the eyepiece port of the microscope, as this has an angle of 45 degrees with respect to the vertical direction. This tilt would provoke the misalignment of the sensor with respect to the exit pupil.

In the Ramsden design, the two lenses can be identical and their separation is equal to approximately two thirds of their focal length. The effective focal length of a two-lens system is:

(6) $$f_{\mathrm{eff}}=\frac{f_{a}f_{b}}{f_{a}+f_{b}-e}.$$

In our case, we chose fa=fb=125 mm, with e=93.75 mm to get an effective focal length of the Ramsden eyepiece of 100 mm. The distances from the lenses to the focal planes are:

(7) $$s_{F^{\prime }}=s_{F}=\frac{1-eP_{a}}{P},$$

where the optical powers are $Pa=\frac{1}{fa}$ and $P=\frac{1}{f\mathrm{EP}}$. From this, we get a total distance between the focal planes of $L=sF+sF^{\prime }+e=143.75$ mm. Compared to the length of a single lens eyepiece (L=2f), we get a gain factor of more than 27%.

**Figure 2:**
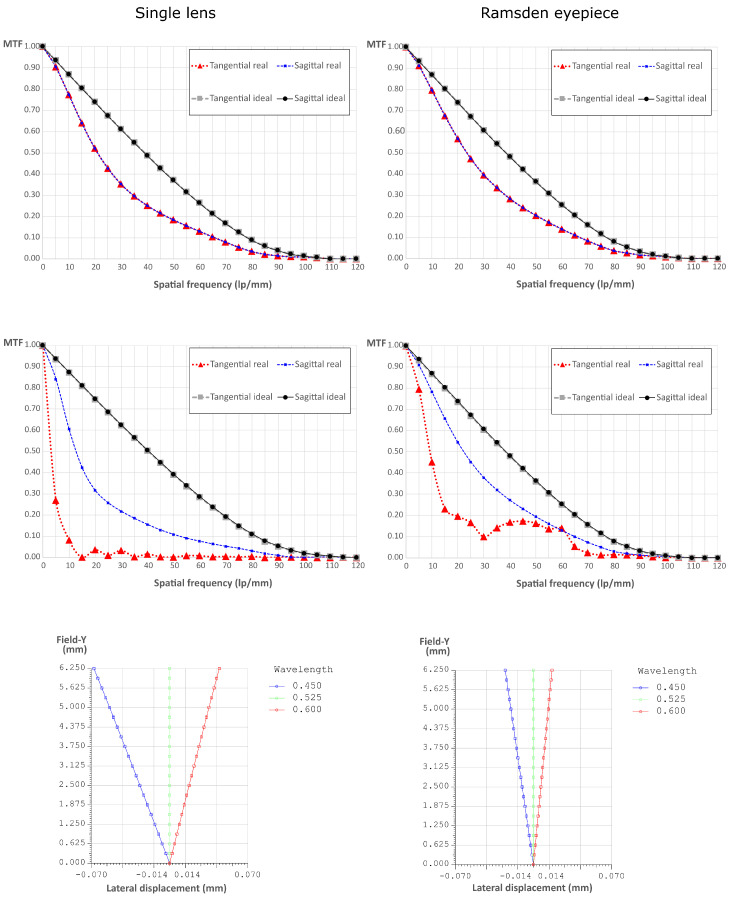
Comparison between two possible eyepiece systems: a single lens and a Ramsden eyepiece. Top line: the MTFs at the center of the FOV. Central line: the MTFs at the edge of the FOV. Bottom line: the lateral color displacement as a function of the lateral displacement in the FOV.

In Figure 2 we show the comparison between the Ramsden eyepiece and a single lens. We simulated both systems with the optical engineering software OpTaliX and we extracted the MTF at the center of the field of view (top line), at the edge of the field of view, HFOV=6.25 mm (central line), and finally the lateral color displacement along the FOV (bottom line). To do so, we supposed the object at infinity and analyzed the image formation [20]. The superiority of the proposed arrangement is evident. In fact, the MTF is closer to the ideal one and the maximum lateral color displacement is more than 2.7 times smaller than for the single lens.

### 2.3. Mechanical Building

**Figure 3:**
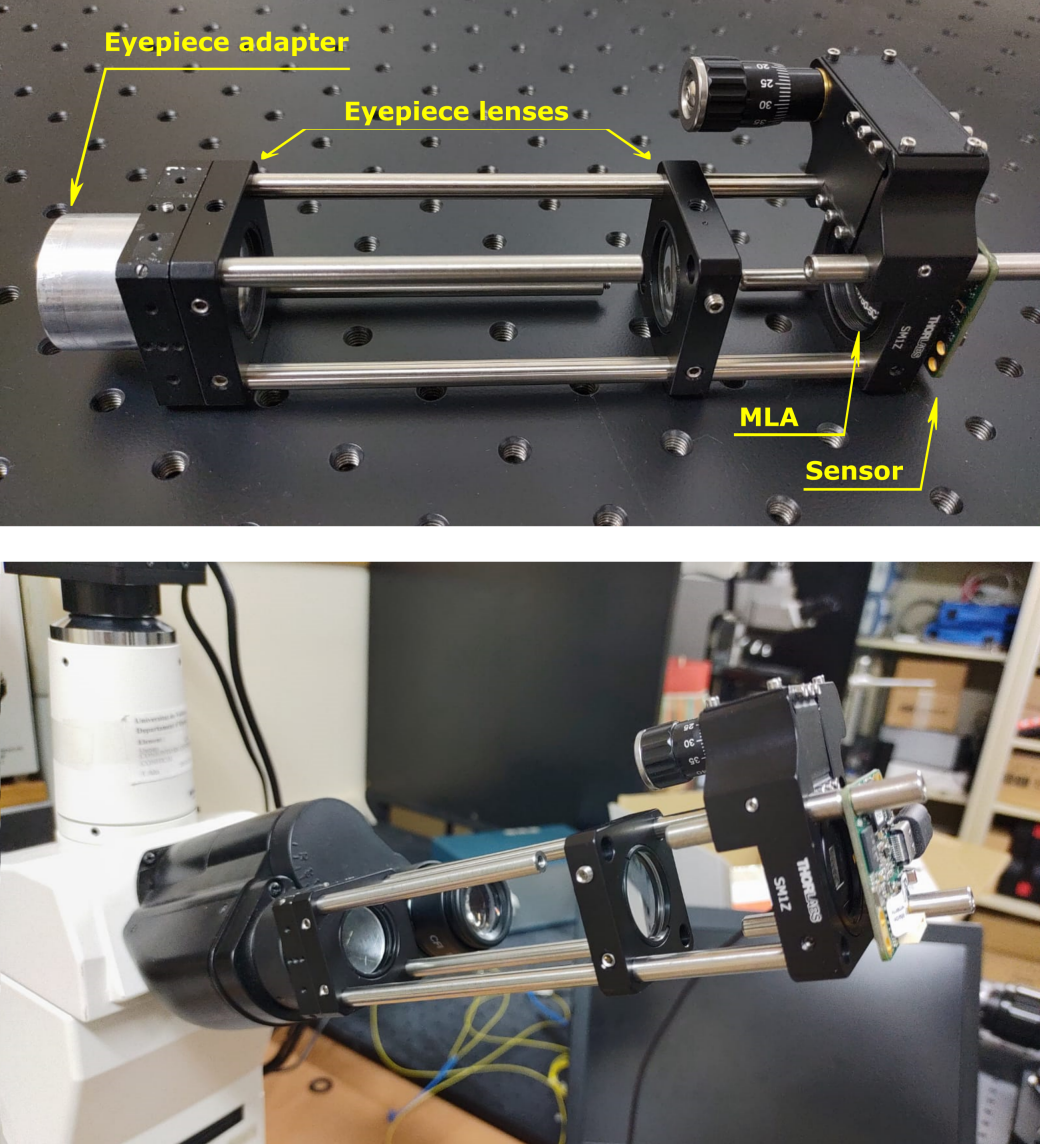
The proof-of-concept device. On the top, the device with all its parts indicated. On the bottom, the device inserted into the microscope.

For the mechanical construction of the device, we used both optomechanical components from Thorlabs and self-built mechanical parts. The device is shown in Figure 3. The components indicated in Figure 3 match those of Figure 1 that lie inside the box marked as “lightfield eyepiece”. The prototype is based on a cage system, so external light could worsen the image quality, above all in fluorescence imaging. In future developments, a closed prototype will be built to avoid this issue.

For coupling the device to the eyepiece port of the microscope, we used a cylindrical adapter whose outer diameter is equal to the inner diameter of the eyepiece port. From the international standard ISO 9345:2019(E), we know that the intermediate image of a microscope is formed inside the eyepiece port at a distance of 10 mm from the eyepiece locating surface of the viewing tube. So, we inserted a 3D-printed field stop into the eyepiece adapter, right at that distance. The MLA was inserted in a 3D-printed holder. This holder was inserted in a Z-axis translation mount with a micrometer knob for fine positioning of the MLA at a distance *f_MLA_* from the sensor.

We had to adjust the distance of the MLA-sensor system to the second lens of the eyepiece. In fact, in the microscope, the objective and the tube lens are not in telecentric configuration, as *d_TL_* < *f_TL_*, where *d_TL_* is the distance between the aperture stop of the microscope objective and the tube lens, and *f_TL_* is the focal length of the tube lens. This produces an axial displacement of the exit pupil from the back focal plane of the eyepiece, which depends on the distance between the objective and the tube lens:

(8) $${l^{\prime }}_{\mathrm{EP}}={\left(\frac{f_{\mathrm{EP}}}{f_{\mathrm{TL}}}\right)}^{2}l,$$

where *l’_EP_* is the distance from the back focal plane of the Ramsden eyepiece to the position of the exit pupil, and $l=f_{\mathrm{TL}}-d_{\mathrm{TL}}$. As the real distance between the objective and the tube lens is unknown and it may vary in every microscope, it is necessary to adjust the position of the MLA to avoid vignetting effect of the exit pupil.

## 3. Results and Discussion

### 3.1. Performance Verification

First of all, we verified the resolution of the lightfield eyepiece itself (out of the microscope), which we call intrinsic resolution. To do so, we placed the USAF 1951 resolution chart (58–198 from Edmund Optics) at the object focal plane of the eyepiece and we measured the contrast at the highest resolution resolvable elements. We consider the frequency at which the MTF is equal to 10% as the limit, because the measurements show good linearity until that point.

**Figure 4:**
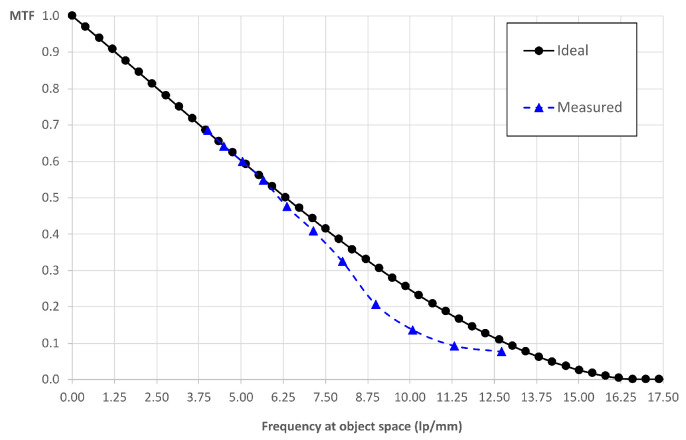
Intrinsic MTF: the ideal and experimental MTF of the lightfield eyepiece when it is not inserted in the microscope.

The overall MTF is the product of two contributions, the diffraction MTF of the optical system and the detector footprint MTF. If we consider an ideal optical system (limited only by diffraction) and a perfect detector, we have an overall ideal MTF [21]:

(9) $$MTF_{\mathrm{id}}=\frac{2}{\pi }\left[cos^{-1}\left(\frac{u}{u_{\mathrm{co},\mathrm{dif}}}\right)-\sqrt{1-{\left(\frac{u}{u_{\mathrm{co},\mathrm{dif}}}\right)}^{2}}\left(\frac{u}{u_{\mathrm{co},\mathrm{dif}}}\right)\right]\times sinc\left(u\delta_{\mathrm{os}}\right),$$

where *u_co,dif_* is the cut-off frequency of the optical system, *u* is the frequency in the object space of the eyepiece and *δ_os_* is the scaled pixel size $\delta_{\mathrm{os}}=\delta$$\frac{f_{\mathrm{EP}}}{f_{\mathrm{MLA}}}$. In the case of the eyepiece, we have $u_{\mathrm{co},\mathrm{dif}}={\left(\frac{\lambda }{2{\mathrm{NA}}_{\mathrm{ML}}}\frac{f_{\mathrm{EP}}}{f_{\mathrm{MLA}}}\right)}^{-1}$≃ 16.67 lp/mm. We get MTF = 10% for u=12.88 lp/mm. In Figure 4, we show the comparison between the ideal MTF and the experimental one. The experimental value of the frequency at which the MTF is equal to 10% is 10.84 lp/mm.

**Figure 5:**
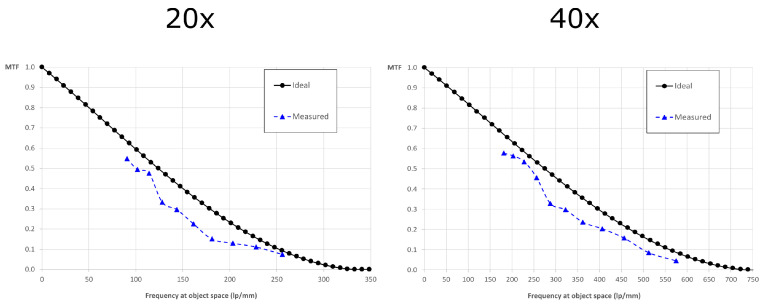
Extrinsic MTFs: the ideal and experimental MTF of the entire system when the lightfield eyepiece is coupled to the host microscope. On the left, the MTF with the 20x objective. On the right, the MTF with the 40x objective.

Once the intrinsic resolution was validated, we verified the extrinsic resolution with the objectives cited above. To do so, we coupled the device to the host microscope and we placed the USAF chart at the object plane. In this case, the cut-off frequency is $u_{\mathrm{co},\mathrm{dif}}={\left(\frac{\lambda N}{2NA_{\mathrm{OB}}}\right)}^{-1}$ and the scaled pixel size is $\delta_{\mathrm{os}}=\frac{\delta }{M_{\mathrm{TOT}}}$. The theoretical values of the frequency at which the MTF is equal to 10% are 252 lp/mm for the 20x objective and 560 lp/mm for the 40x objective. As we can see from Figure 5, the experimental values are 238 lp/mm and 502 lp/mm. This corresponds to a resolution of 4.20 µm for the 20x objective and 1.99 µm for the 40x objective.

Subsequently, we evaluated the extrinsic depth of field (DOF) with both objectives. To do so, we imaged the USAF resolution chart and we shifted it from the object plane by a constant step. We fixed a frequency (an element of the USAF chart) and we measured the contrast at that frequency at every shifted plane. The DOF is defined as the interval in which the contrast loss is smaller than a factor of $\sqrt{2}$. The theoretical DOF for the FiMic system is:

(10) $$DOF=\lambda {\left(\frac{N}{{NA}_{\mathrm{OB}}}\right)}^{2}+\frac{\delta }{M_{\mathrm{TOT}}}\frac{N}{NA_{\mathrm{OB}}}.$$

**Figure 6:**
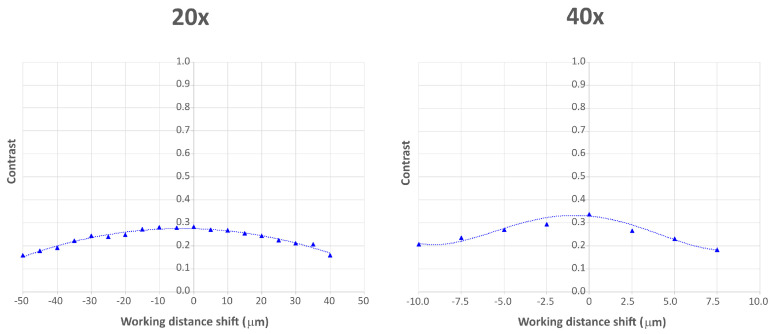
Contrast values over working distance shift for both 20x and 40x objective. The triangles are the experimentally measured values, the dotted lines are the trend lines. The working distance shift has a value 0 when the USAF chart is at the object plane, it has negative values when the USAF chart is moved further from the objective and positive values when the USAF chart is moved closer to the objective.

Substituting the data of both objectives in the equation, we obtain DOF = 73.9 µm with the 20x objective and DOF = 15.0 µm with the 40x objective. To measure the DOF experimentally, we placed the USAF chart at the object plane of the 20x objective, then we displaced it by a constant step of 5.0 µm approximately in both axial directions. We took a picture at every plane and we measured the contrast at a fixed frequency of 143.7 lp/mm (element 7.2 of the USAF chart). With the 40x objective, we displaced the USAF chart by a constant step of 2.5 µm approximately and we measured the contrast at a fixed frequency of 287.4 lp/mm (element 8.2 of the USAF chart). We assumed an error of ±0.5 µm in the shifting, as the axial displacement was made through a manual translation stage. In Figure 6 we show the contrast values over the working distance shift for both objectives. From the experimental values, we extracted the trend line of the contrast. The measured contrast value at the object plane is $c_{\max}$ = 0.2825 and $c_{\max}$ = 0.3368 for the 20x and the 40x objective, respectively. If we solve both trend line equations, calculating the values of the working distance shift at which $c=\frac{c_{\max}}{\sqrt{2}}$, we find that DOF = 72.92 µm for the 20x objective, and DOF = 16.77 µm for the 40x objective. As for the resolution, the experimental values are very close to the theoretical ones.

### 3.2. Sample Imaging

After having verified the 2D resolution of the device, we tested its ability to reconstruct three-dimensional samples. For this purpose, we imaged some thick samples in different host microscopes. The purpose of imaging different samples in different microscopes was to test the functioning of the device when inserted in multiple host microscopes as well as demonstrating its imaging capability. The performances of the device in the different setups depend mainly on the resolution obtained (Equation (5)) and the quality of the microscope objective used. Nevertheless, a comparison of the performances in the different host microscopes is out of the scope of this paper.

As we mentioned in Section 2.3, the distance between the microscope objective and the tube lens is unknown. Hence, the axial position of the exit pupil changes from one microscope to another. Therefore, we had to adjust the position of the MLA to match it with the position of the exit pupil when we inserted the eyepiece into a new microscope, in order to avoid vignetting effects. This was the only necessary adjustment when we changed from one host microscope to another.

To reconstruct the sample volume, we used the shift and multiply (S&M) method [22], which is capable of providing optical sectioning in real time. Furthermore, this algorithm avoids the background noise typically provided by deconvolution methods. The axial thickness of the optical sections depends on the parameters of both the device and the host microscope:

(11) $$\rho_{z}=\frac{\delta }{{M^{2}}_{\mathrm{TOT}}}f_{\#,\mathrm{MLA}}.$$

**Figure 7:**
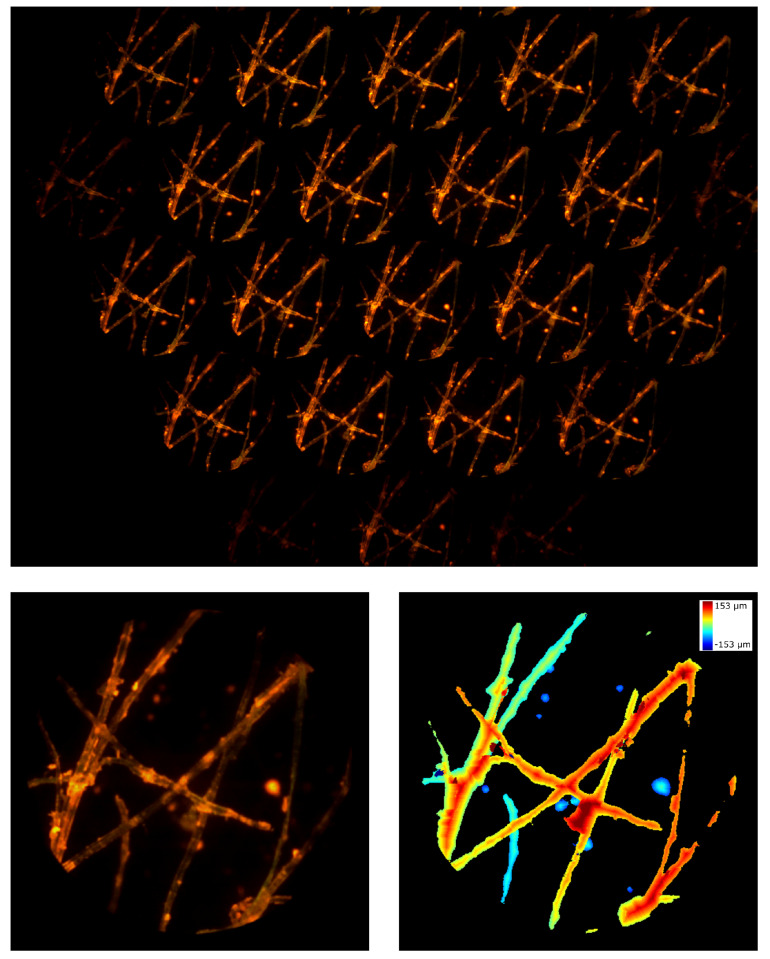
Cotton fibers. On the top, the entire frame registered by the device. On the bottom, the central perspective view and the depth map calculated from the lightfield image.

Firstly, we used the microscope described above to image cotton fibers with the 20x objective. The fibers were stained with a fluorescent highlighter so that the emission could be observed with a filter cube with these data: excitation filter EX510-560, dichroic mirror DM575, barrier filter BA590. The results are shown in Figure 7. In the entire frame (top image), the sensor is not uniformly illuminated due to a slight tilt of the device when it is inserted into the eyepiece port of the Nikon TE2000-U microscope. In addition, some portions of the sample are defocused. This is because the total depth of the sample is greater than the depth of field of the system. Nevertheless, we were capable of reconstructing the entire sample. In Video S1 of Supplementary Materials, we show the focal stack calculated from the lightfield image. Here, the axial thickness of the sections is $\rho_{z}=$ 6.9 µm and the total depth of reconstruction is 305.5 µm.

**Figure 8:**
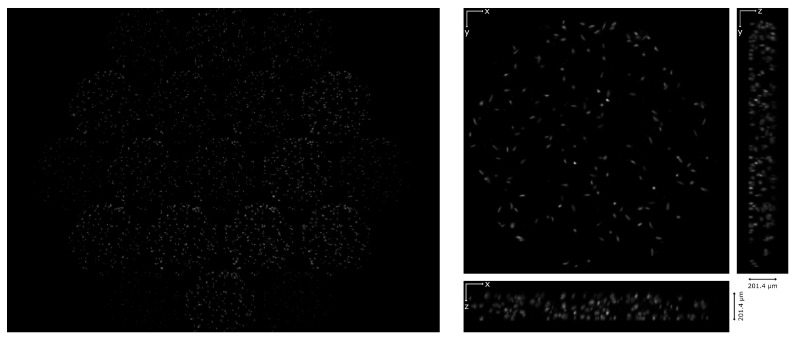
Living phytoplankton. On the left, the first frame of the video recorded through the lightfield eyepiece. On the right, the corresponding reconstruction: the Z projection and the orthogonal views are shown.

Then, the device was tested in an Amscope microscope (T670Q-PL-FL). Living phytoplankton were imaged using darkfield illumination and a 20x/0.40 objective. We recorded a video of the moving sample and then reconstructed it in a volume of 201.4 µm of depth, with $\rho_{z}=$ 6.9 µm. The video recorded through the device and the reconstruction are shown in Video S2 and Video S3 of Supplementary Materials, respectively. In Figure 8 the first frame of the video and its corresponding reconstruction are shown.

**Figure 9:**
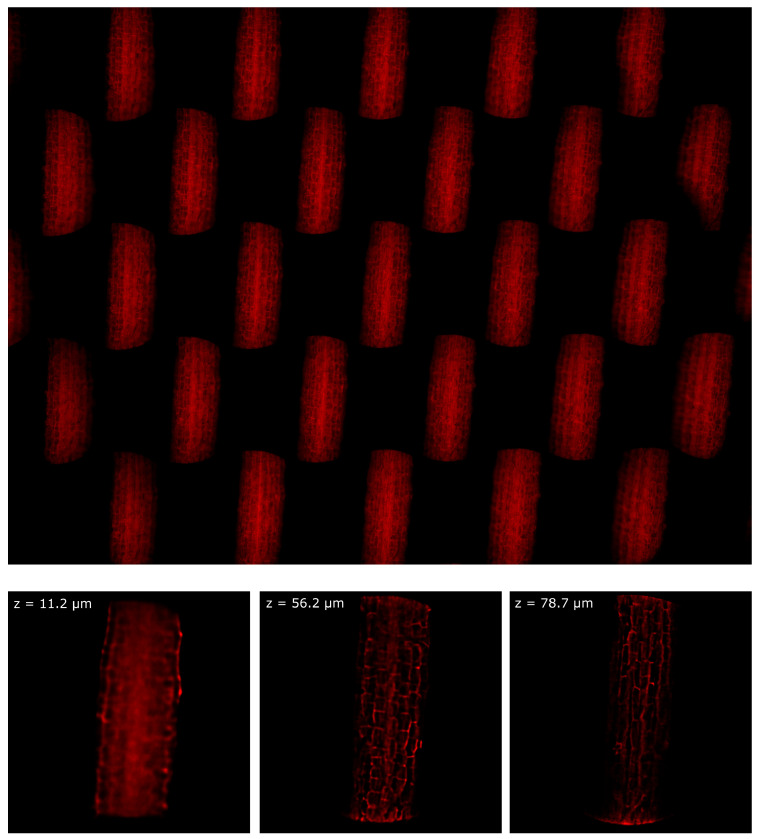
Hypocotyl of Arabidopsis Thaliana imaged with a water immersion objective. On the top, the entire frame registered by the device. On the bottom, the sample reconstructed at three different depths.

Finally, the device was tested in a Leica TCS LSI microscope. In this case, the sample was Arabidopsis Thaliana stained with Propidium Iodide. The sample was prepared as described in [23] and it was observed in water immersion with a 20x/1.0 Olympus objective. In Figure 9 we show one picture of the hypocotyl of the plant and three images of the sample at three different depths of reconstruction. The entire focal stack is shown in Video S4. In this configuration, $\rho_{z}=$ 5.6 µm, because the effective magnification of a 20x Olympus objective mounted in a Leica microscope is $M_{\mathrm{OB},\mathrm{eff}}=22.2x$, because of the different focal length of the tube lens. Despite the intrinsic loss of resolution of the lightfield system, we can still see the cell walls with good contrast.

## 4. Conclusions

In this paper, we presented the lightfield microscope eyepiece, which converts any optical microscope into a lightfield microscope. We detailed the choice of the optical and mechanical components and we demonstrated its functioning with multiple configurations, varying the host microscope, the objective, and the illumination technique. We demonstrated that the overall system achieves 1.99 µm lateral resolution at 10% contrast with a 40X objective and 4.20 µm lateral resolution at 10% contrast with a 20X objective, in line with the theoretical performances. Finally, we imaged different thick samples and demonstrated the ability of the system to reconstruct volumes of up to 300 µm of depth.

## 5. Patents

The lightfield microscope eyepiece is a patented device (publication number WO/2020/ 030841) [24] and it is developed and commercialized by Doitplenoptic S.L. [25].

## Data Availability

The data presented in this study are contained within the article and the Supplementary Materials.

## Supplementary Materials

The following are available online at https://www.mdpi.com/article/10.3390/s21196619/s1, Video S1: Focal stack of the fibers sample, Video S2: Lightfield video of the living phytoplankton, Video S3: Volume reconstruction of the living phytoplankton, Video S4: Focal stack of the Arabidopsis Thaliana sample.
